# Computer-Guided Evaluation of the Use of Two Different Devices in the Reduction of Inferior Tooth Crowding

**DOI:** 10.3390/clinpract14030094

**Published:** 2024-06-20

**Authors:** Sara Di Nicolantonio, Maria Ausilia D’Angelo, Davide Pietropaoli, Annalisa Monaco, Eleonora Ortu

**Affiliations:** Dental Unit, MeSVA Department, University of L’Aquila, P.le S. Tommasi, 67100 L’Aquila, Italy; saradinicolantonio@libero.it (S.D.N.); mariaausilia.dangelo@student.univaq.it (M.A.D.); davide.pietropaoli@univaq.it (D.P.); annalisa.monaco@univaq.it (A.M.)

**Keywords:** elastodontic device, tooth crowding, orthodontic treatment, clear aligners

## Abstract

Objective: Lower tooth crowding is considered one of the most common malocclusions in growing patients and due to the potential complications associated with it, it is recommended to intercept this condition as soon as possible. The purpose of this paper is to examine and compare the effectiveness of two different orthodontic devices (elastodontic device and clear aligners) in the treatment of anterior tooth crowding in the jaws of young patients. Materials and methods: Seventy patients aged between 10 and 16 years with anterior inferior tooth crowding were recruited into this study and divided into case and control groups. The former group comprised 35 patients (15 males and 20 females, average age 10.2 years) who were treated with elastodontic devices (EQ CP series, Eptamed), while the control group consisted of 35 patients (15 males and 20 females, average age 10.5 years) who received aligners (Invisalign). All patients underwent periodic visits after 6 months from the start of treatment (T1) and after 1 year (T2) in which the progress of therapy was evaluated by measuring the inferior intercanine distance using a digital caliper. A parametric ANOVA test was conducted for statistical analysis. Results: There is no statistically significant difference between the two groups at either T1 or T2 (*p* < 0.05), thus making the two treatment modalities comparable. Conclusions: Both elastodontic devices and aligners can be considered as effective tools to successfully conduct inferior expansive treatment for the resolution of tooth crowding; however, the elastodontic devices are considered more comfortable to wear and they are required to be worn for less time during the day.

## 1. Introduction

Mandibular anterior crowding is one of the most frequent malocclusions in the Caucasian population, due to a reduction of the intercanine distance in the mandible. Mandibular anterior crowding is identified as the discrepancy between the mesiodistal width of the teeth of the four permanent incisors and the space available in the alveolar process. According to a recent meta-analysis [1], it can worsen with age and with the development of permanent dentition. While in deciduous dentition only 16% of the overall population has tooth crowding, in mixed dentition, the prevalence rises to 37% and up to 39% in permanent dentition. To date, the causes leading to the development of this malocclusion remain uncertain. An undersized mandibular bone or oversized teeth are just some of the factors related to anterior tooth crowding. In addition, with aging, due to various physiological processes, there is a progressive reduction in the size of the jawbone, which affects the amount of space available for the placement of dental elements in the arch. This inevitably has a negative effect on the intercanine distance [2]. It will, in fact, decrease, causing tooth crowding from mild to more severe situations, which may even require tooth extractions. This significant decrease in length of the lower arch is linked to caudal cranial growth, tooth permutation, and the consequent mesialisation of the first molars due to loss of space [3]. Other authors associate tooth misalignment with the eruption of the lower third molars [4]. The current opinion is that third molar eruption in the arch may contribute to the presence and extent of crowding by preventing posterior teeth from moving distally, inevitably leading, due to differential growth, to the development of crowding [5]. However, to date there is no scientific certainty about this. On the other hand, a recent study found among various possible associations and etiopathogenic hypotheses of lower dental crowding, a significant correlation between the morphology of the sella turcica, dental abnormalities, anteroposterior skeletal pattern, and microdontia [6]. Tooth crowding can be also a risk factor for the development of interproximal caries and periodontal disease. The lack of space between teeth can make oral hygiene manoeuvres more difficult, especially flossing, promoting plaque build-up and bacterial proliferation, mechanisms underlying periodontal disease [7]. In this regard, today it is necessary to intercept the lack of space in the dental arches early on to prevent it from worsening in childhood. A therapeutic course of orthodontic treatment may be a good option for dental realignment, aimed not only at improving aesthetics or function but also at preventing the progression of periodontitis [8]. Anterior tooth crowding treatment techniques can be various; the most successful of these are lingual arches, which act by favouring the maintenance of space in the arch, and Schwartz’s plate, which, following the activation of a screw, induces the dento-alveolar expansion of the mandible, increasing the interarch space, as well as the various fixed orthodontic techniques and dental extractions that are carried out in cases where tooth crowding is very severe. Among these, removable appliances such as silicone elastodontic devices and transparent masks have recently been introduced [9].

However, according to a recent study, the degree of tooth crowding influences the type of treatment to be undertaken [10]. While in more complex degrees of crowding most orthodontists prefer an extractive procedure of the first or second premolar, in cases of mild crowding, a less invasive, hence non-extractive, technique is preferred. However, the number of cases considered extractive changes drastically depending on the method used to assess the degree of crowding. In fact, the use of digital instruments, such as intra-oral scans, allows the orthodontist to truly assess the severity of crowding, avoiding the overestimates that can occur with direct visualisation, which would lead the orthodontist to extractive treatment where it is not necessary [10].

In recent years, various types of orthodontic devices have been introduced on the market that can improve and in some cases resolve this malocclusion, switching from fixed orthodontic therapy to removable therapy with the use of clear aligners or elastodontic devices. The latter are preformed removable silicone devices, which therefore do not require construction in the laboratory. They are able to act not only at a dental level but also at an orthopaedic level, acting on different types of malocclusion and guiding the correct positioning of the teeth in the arch [11].

These devices are widely used in clinical practice due to their elastic shape and consistency, which makes them pleasant and comfortable for the patient. The main objective of Eptamed balancers is to restore the stomatognathic functions of swallowing and breathing of the small patient, eliminating any vicious habits.

On the other hand, clear aligners are widely used and requested orthodontic devices by patients. They allow tooth movement by placing composite attachments on the tooth surface while respecting the patient’s aesthetics and avoiding the use of brackets and wires [12].

Both elastodontic devices and clear aligners are capable of acting in different types of malocclusions, including anterior tooth crowding. Although there are countless treatment methods for the resolution of inferior tooth crowding, the authors focused on these two methods because they are considered to be among the most innovative and in demand on the current market. It is therefore considered necessary to assess their validity and clinical effectiveness. The objective of the aforementioned study is to compare the use of two different devices (EQ Series CP (Eptamed), Via Ravennate, 979, 47522 Cesena (FC), ITALY versus Invisalign, 410 North Scottsdale Road, Suite 1300 Tempe, Arizona 85288) for the treatment of mandibular anterior tooth crowding by assessing the intercanine distance, before, after 6 months of treatment, and at 1-year follow-up.

## 2. Materials and Methods

### 2.1. Study Population

This study was carried out in accordance with the fundamental principles of the Declaration of Helsinki. It was approved before commencement by the Ethics Committee of the University of L’Aquila, Italy (57/2021-22), on 21 December 2021. A total of 120 patients aged 10–16 years were examined in the Dental Clinic of the University of L’Aquila. Each patient underwent several diagnostic examinations: extra-oral and intra-oral photos, an orthopantomography, a latero-lateral teleradiography, and intra-oral scans of the arches for each patient were taken. Following the case study, the same orthodontist drew up a customised treatment plan for each patient based on the Indices of Orthodontic Treatment Need (IOTN) [13]. The degree of tooth crowding was assessed by measuring the inferior intercanine distance with a digital caliper (Sylvac, Fowler, OPTO-RS232 SIMPLEX/DUPLEX, Sweden). It was positioned at the best estimate of a line bisecting the incisor segment in the mandibular arch. Three measurements were taken for each distance and their average was used as the final value. The test population was then selected according to the exclusion and inclusion criteria listed below.

Exclusion criteria:

- IOTN index >4;
- Congenitally missing permanent teeth or premature loss of deciduous or permanent teeth;
- Previous orthodontic treatment;
- Presence of epilepsy;
- Systemic disease;
- TMD;
- No written informed consent from a parent or legal guardian.

Inclusion criteria:

- Skeletal Class I relationship (ANB 2° +/− 2°);
- Molar Class I relationship;
- Eruption of permanent lower incisors and canines;
- Presence of moderate lower tooth crowding;
- SNA angle between 82° +/− 2 °;
- SNB angle between 80° +/− 2 °.

**Figure 1:**
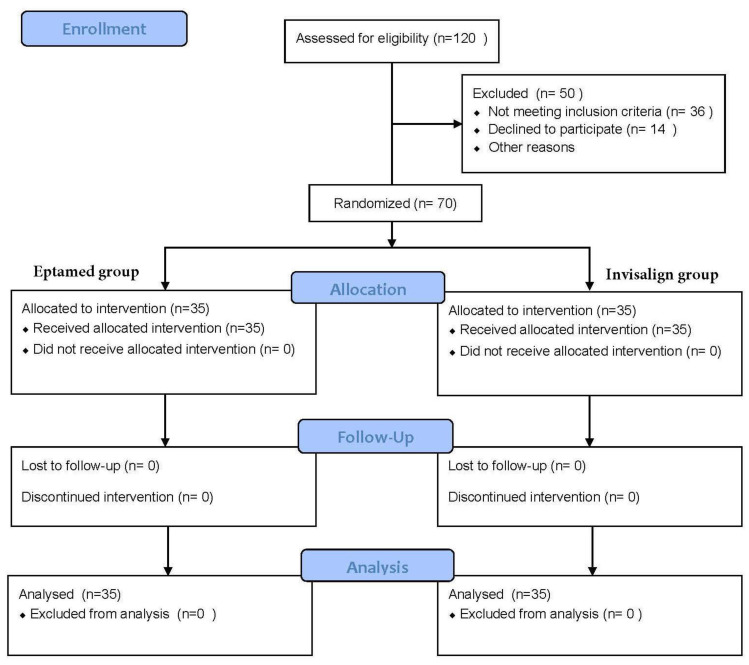
Flow diagram of the progress of the two groups through the phases of this study.

As described in Figure 1, of the 120 patients initially enrolled, 50 patients were excluded from the study because they did not meet the inclusion criteria or did not give consent to participate. In the end, 70 patients were considered eligible and were divided into two groups (case group and control group) with the use of online randomisation software (https://www.sealedenvelope.com/ (accessed on 13 January 2022)). The demographic characteristics of the two groups are described in Table 1 below. The authors preferred to choose patients between the ages of 10 and 16 years at the end of orthopaedic functional therapy, in order to allow all dental elements to fully erupt in the arch. Moreover, they were required to be highly cooperative. As the devices were removable, patients were instructed to wear them for as long as possible during the day, to manage personal and device oral hygiene, but, above all, to stick to the treatment plan (e.g., changing the clear aligners every week in the Invisalign group). The case group consisting of 35 patients, 15 males and 20 females with an average age of 10.2 years (SD = 1.5), was treated with the Eptamed balancer, while the control group consisting of 35 patients, 15 males and 20 females with an average age of 10.5 years (SD = 1.4), was treated with Invisalign aligners. The degree of anterior inferior tooth crowding was calculated by considering the inferior intercanine distance, which was evaluated with a digital caliper via iTero intra-oral scan software at baseline (T0), 6 months after the start of treatment (T1), and 1 year after treatment (T2) by the same orthodontist. These variables were analysed using an ANOVA test. Our null hypothesis is that there are no statistically significant differences between the two groups.

### 2.2. Experimental Settings

**Figure 2:**
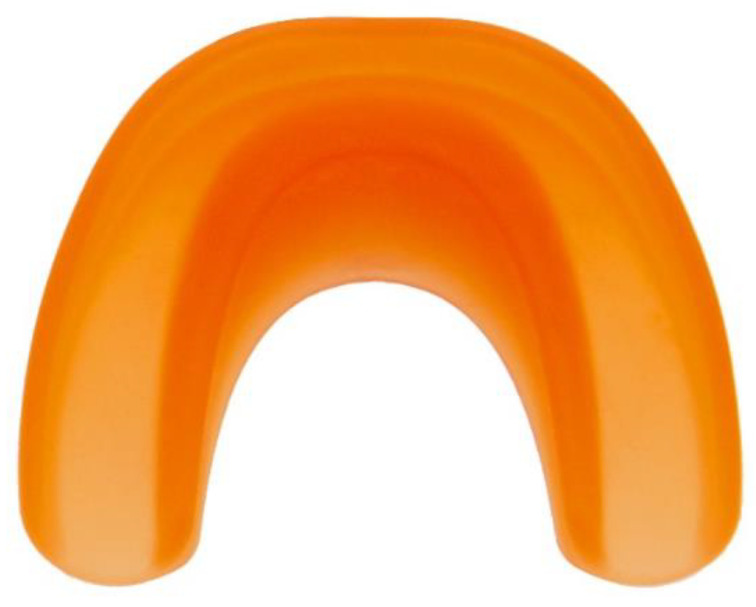

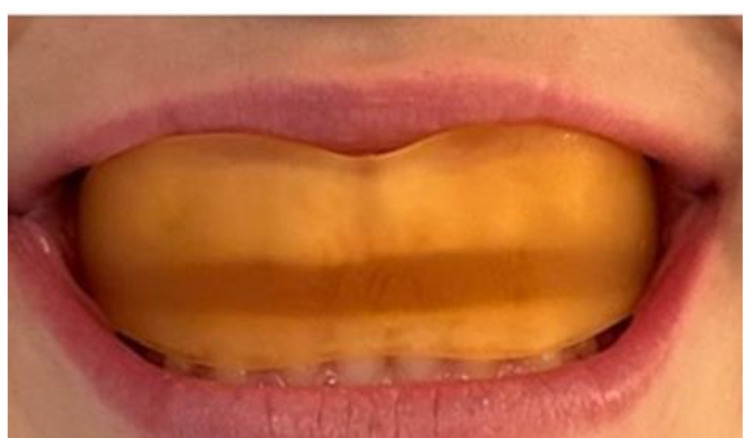
Examples of an Eptamed EQ Series CP device.

The case group patients received an Eptamed EQ Series CP (Via Ravennate, 979, 47522 Cesena (FC), Italy) device of medium hardness (Figure 2). The Eptamed elastodontic devices are similar to a mouth guard, of different hardness and size, and they cover both arches up to the last molars. The size of the device was chosen for each patient by calculating the distance between the palatal cusps of the first premolars or deciduous molars on the scans taken at the first visit. It was worn every night and for two hours a day. During daytime, the patient had to perform exercises consisting of chewing on the device. Chewing the device balances the tension at the level of the sphenobasilar synchondrosis, according to the principles of osteopathic medicine and philosophy [14]. The elastic consistency and the shape of the device act on the altered functions of the mouth, facilitating the correct position of the tongue and favouring nasal breathing. It relaxes the oro-facial musculature while transmitting the necessary input for the correct positioning and alignment of the teeth in the arch. Patients wearing the balancer were visited by the same orthodontist every month to assess the progress of therapy. During these visits, changes were made to the device if necessary and every 4 months the patient received a new device of greater hardness and of a size appropriate for the growing arch. The patients in the control group received clear aligners for both arches to be changed every week until the end of therapy. They had to wear these devices as long as possible during the day and throughout the night, while the clear aligners could only be removed during meals. Patients in both groups were monitored after 6 months and after 1 year from the start of treatment in which the effectiveness of the two devices was investigated by calculating the lower intercanine distance by means of a digital caliper. All measurements were made by the same operator. To assess the reliability and reproducibility of the measurement technique, a combined error of position, tracking, and landmark measurement was determined. The method error was calculated from the double measurement of 30 randomly selected dental models, measured again after an interval of 1 week, using Dahlberg’s formula. The lower anterior tooth crowding was treated by using the available leeway space and performing a slight proclination of the anterior–inferior tooth sector. However, all patients were followed over a year until the desired result was achieved; moreover, some of these patients required fixed orthodontic therapy for minor finishing touches.

All patients complied with the treatment protocol and none of them refused treatment.

## 3. Results

The baseline conditions of the recruited individuals revealed no statistical differences in terms of age and gender. This enabled the two groups to be considered comparable. Data normality was tested by the Shapiro–Wilk test. A parametric approach was then used for differential statistics. Analysis of variance (ANOVA) was used for comparisons between groups. Statistical significance was set at *p* < 0.05.

**Figure 3:**
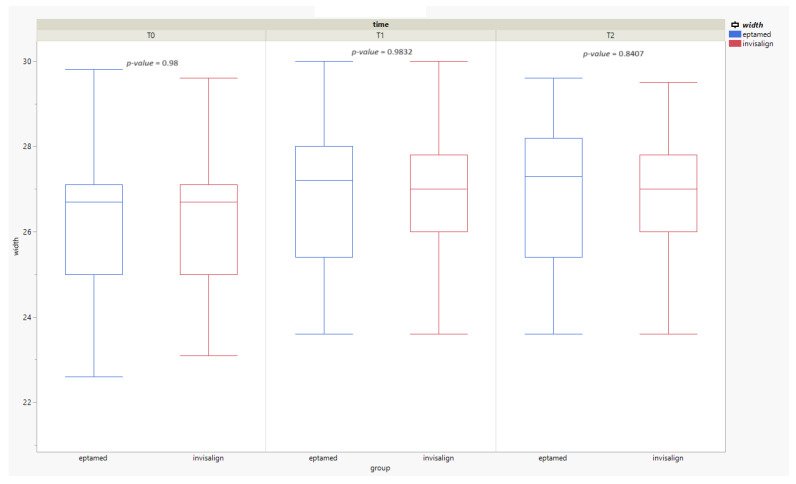
Box plot of the lower ICW values time-stratified for the “Eptamed” group vs. the “Invisalign” group. The x-axis represents the two groups (Eptamed and Invisalign) in the three treatment phases (T0, T1, and T2) and the y-axis represents the intercanine distance expressed in millimetres (width). The two groups had the same results: T1 (*p* = 0.9832) and T2 (*p* = 0.8407).

There were no statistically significant differences between the test and control groups at T0, T1, or T2. As shown in Table 2 and Figure 3, the two appliances were found to be equally effective at T1 (*p* = 0.9832) and T2 (*p* = 0.8407) in widening the distance between the lower canines. This confirmed the authors’ assumptions on the comparative effectiveness of the two devices. Statistical analysis and plots were generated by SPSS Statistics 29.0.2.0. 

## 4. Discussion

**Figure 4:**
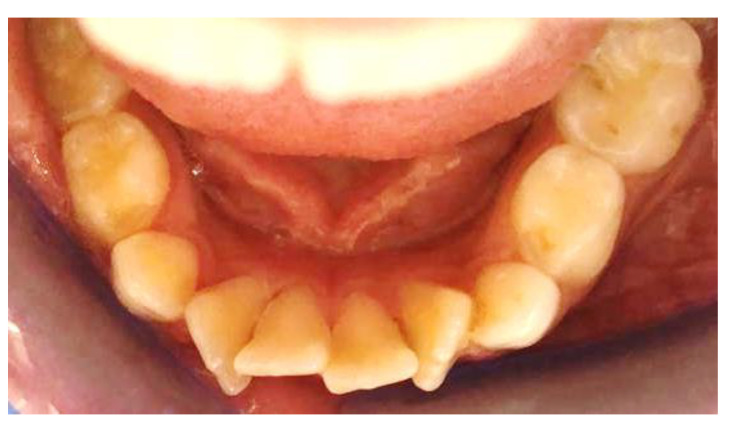
Intra-oral photos before in a patient treated with Eptamed for inferior anterior crowding.

**Figure 5:**
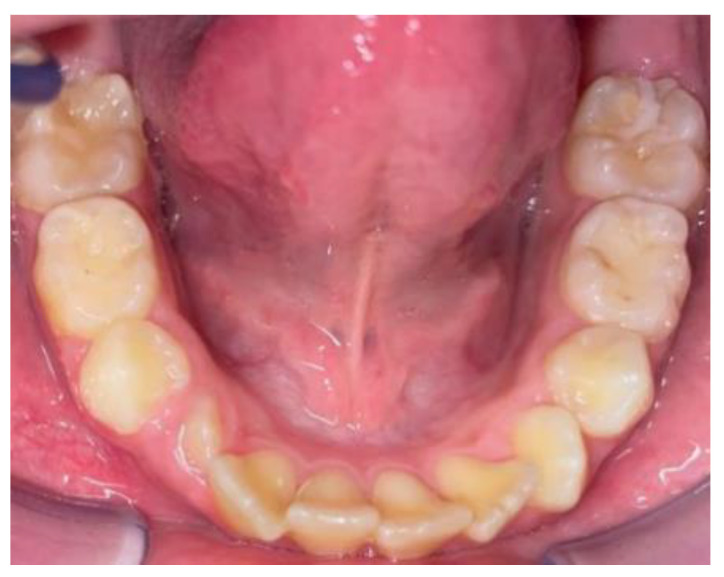
Intra-oral photos after treatment in a patient treated with Eptamed.

**Figure 6:**
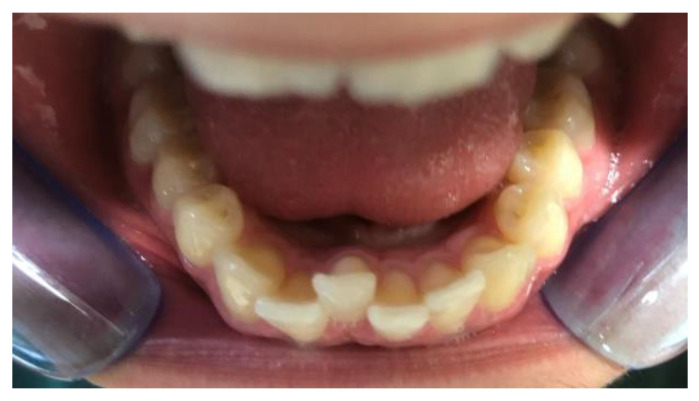
Intra-oral photos before after the start of treatment with Eptamed for inferior anterior crowding.

**Figure 7:**
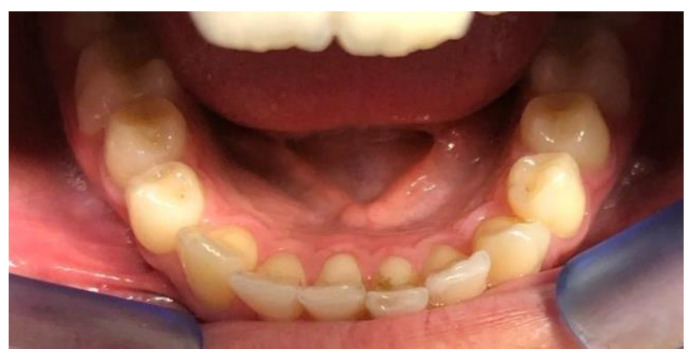
Intra-oral photos after treatment with Eptamed.

**Figure 8:**
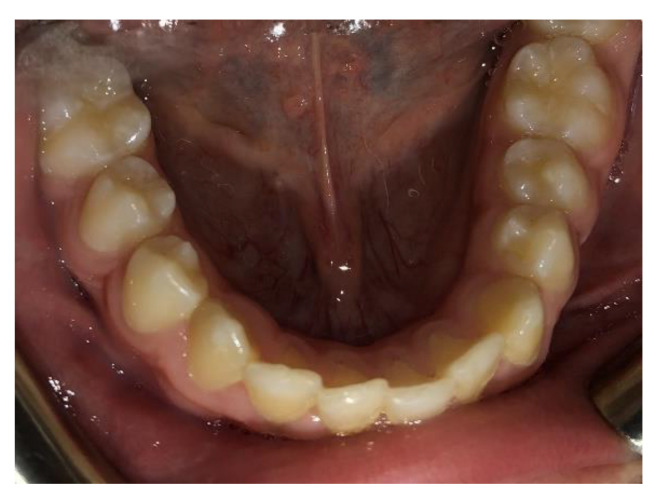
Intra-oral photos before in a patient treated with Invisalign for inferior anterior crowding.

**Figure 9:**
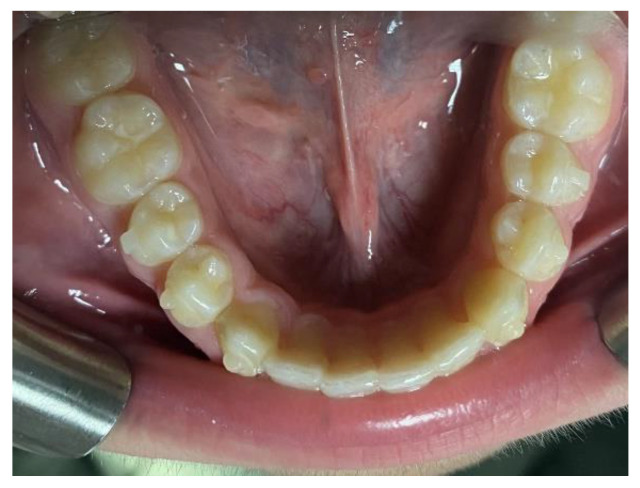
Intra-oral photos after treatment with Invisalign.

**Figure 10:**
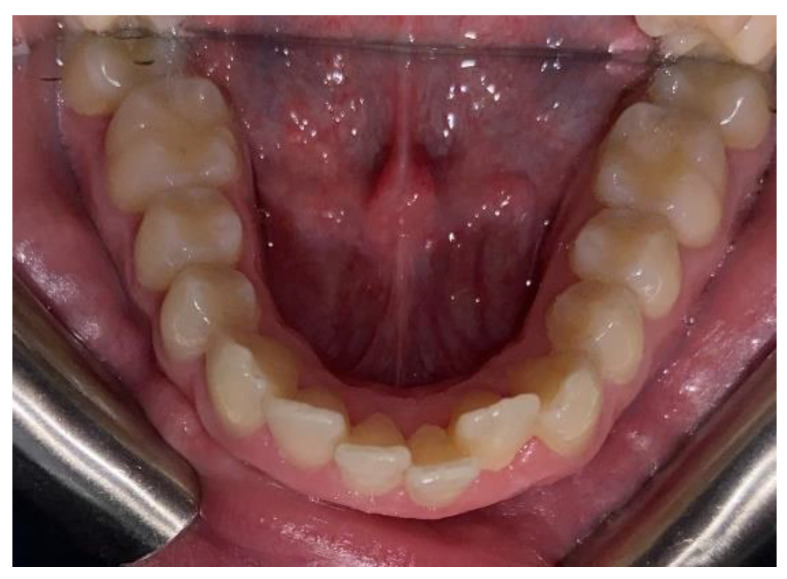
Intra-oral photos before in a patient treated with Invisalign for inferior anterior crowding.

**Figure 11:**
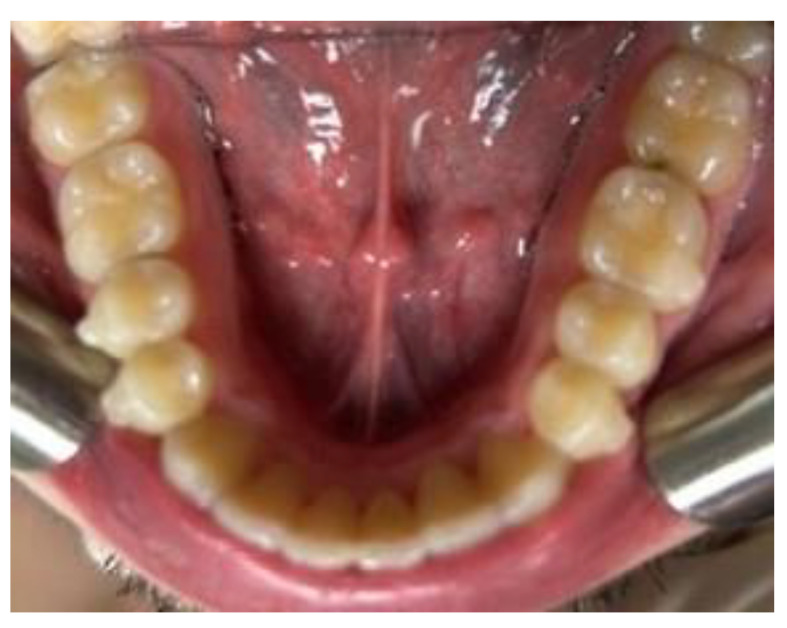
Intra-oral photos after treatment in a patient treated with Invisalign for inferior anterior crowding.

From the results observed, it may be concluded that both the Eptamed elastodontic device and the clear aligners are equally effective in the treatment of lower tooth crowding., We conclude that the two therapies analysed are similar in terms of treatment time, success rate, reliability, adoptability, and post-treatment recurrence (after careful evaluation of wisdom teeth). There is a superiority of one device over another in terms of cost (Eptamed is less expensive). In fact, after 6 months from the beginning of the treatment and after 1 year, there were no significant differences; both devices act by increasing the intercanine distance, causing an expansion of the lower arch (Figure 4, Figure 5, Figure 6, Figure 7, Figure 8, Figure 9, Figure 10 and Figure 11). Aligners, being transparent and therefore aesthetic devices, have been gaining great acceptance in recent years among orthodontists but especially among patients. Moreover, the use of digital aids, such as intra-oral scanners, make it possible to previsualise the final result following treatment with aligners, increasing demand. However, according to a recent systematic review, the virtual results predicted by digital software may be overestimated compared to the actual achievable clinical results. For this reason, the orthodontist’s role in adjusting and planning tooth movements based on his or her clinical experience is important [15].

There are conflicting results in the literature regarding the effectiveness of aligners in orthodontics. The most predictable tooth movement is tipping in the bucco-lingual direction; on the other hand, mesio-distal rotational movements are the most difficult to achieve, as intrusion and extrusion movements [15].

With regard to the resolution of inferior tooth crowding, clear aligners are capable of acting, especially in cases of mild-to-moderate misalignment. Up to approximately 6 mm, the alignment system with such devices was, in fact, able to produce clinical results comparable to digital set-up planning [16]. However, aligners act on tooth crowding by tilting only the crown but not the tooth root; while aligners can improve aesthetics, comfort, and dental hygiene, there is reduced control over some tooth movements [17]. Moreover, the predictability of resolving crowding with aligners is greater in the upper arch (87%) than in the lower arch (81%) [18]. Of the various strategies for gaining space in the jaw with clear aligners, changing the inclination of the incisors seems to be most effective, while the least reliable is an increase in the transverse diameters of the lower jaw [19]. The predictability of resolving crowding with aligners depends on several factors: the clinical experience of the orthodontist, the shape and position of the attachment, and the material and thickness of the aligner [20]. Moreover, a single treatment plan may not be sufficient, so in the vast majority of cases, additional aligners must be considered to resolve the present crowding.

Elastodontic devices can be considered valid alternatives for the treatment in question. They are able to act by increasing the intercanine distance as well as clear aligners. Unlike the latter, however, they are worn for a few hours during the day, which may have a positive effect by increasing patient compliance. The consistency of the material from which they are made also prevents injuries to soft tissue and mucous membranes.

Elastodontics is in all respects a minimally invasive, interceptive orthodontic therapy, so much so that according to a recent study by Ortu et al. [21], the use of such devices allows for greater muscle relaxation in patients with mandibular retrusion. Such devices are also able to reduce muscle tension in patients with temporomandibular disorders, thus decreasing the risk of worsening symptoms during orthodontic treatment itself [20,21].

In addition, patients with Class II malocclusion and oral respirators, with the use of elastodontic appliances, have achieved an increase in the size of the upper airway, increasing the passage of air from the nostrils as opposed to the oral cavity. This results in an improvement of not only respiratory but also masticatory and phonatory activity [22].

According to the review by Migliaccio et al. [23], the scientific literature to date is in agreement in claiming excellent results in the treatment of lower tooth crowding with these devices. They are capable of acting by expanding the mandibular arch and consequently creating space to increase the intercanine distance.

However, ours is a pilot study with several limitations. Indeed, we considered growing patients with a very precise age range and they were evaluated for an unfortunately limited period of time. In order to overcome these drawbacks, it would be optimal to perform a large-scale longitudinal study, thus increasing the study sample size and follow-up period. Only in this way can the effectiveness of elastodontic devices and clear aligners in lower tooth crowding be truly established with certainty.

## 5. Conclusions

Elastodontic devices as clear aligners can be valid options in the treatment of anterior tooth crowding. In fact, they have achieved results that are quite similar with an increase in the intercanine distance. Moreover, according to the authors, elastodontic devices could be more comfortable for the patient by being worn only during the night and for two hours a day. In addition to acting orthodontically, they are also capable of acting on the functions of the stomatognathic system, improving breathing and swallowing, a peculiarity that clear aligners do not possess. However, further studies will be necessary in the future to supplement our knowledge in this respect. In particular, it would be appropriate to pursue longitudinal studies, with a larger sample size and possibly other age groups to fill the gaps in the above-mentioned study.

## Figures and Tables

**Table 1:** Baseline characteristics of the two groups.

Stratified by Treatment			
	Eptamed	Invisalign	*p*-Value
***Sample***	**35**	**35**	
***Sex*** **= M (%)**	15 (42.8)	15 (42.8)	1000
***Age*** **(mean (SD))**	10.2 (1.5)	10.5 (1.4)	0.18
**SNA (mean (SD))**	82.3 (1.06)	82.5 (0.92)	0.39
**SNB (mean (SD))**	80.7 (0.94)	80.9 (0.90)	0.09
**ANB (mean (SD))**	1.28 (0.59)	1.36 (0.59)	0.12

**Table 2:** Mean and standard deviation (SD) of lower ICW values stratified by timing and group.

	T0	T1	T2
**EPTAMED** (lower ICW, (mean (SD)))	26.27 (1.79)	26.85 (1.69)	27.0 (1.83)
**INVISALIGN** (lower ICW, (mean (SD)))	26.21 (1.71)	26.78 (1.66)	26.9 (1.74)

## Data Availability

The data presented in this study are available on request from the corresponding author. The data are not publicly available due to privacy.
